# Development of kinomic analyses to identify dysregulated signaling pathways in cells expressing cytoplasmic PrP

**DOI:** 10.1186/1743-422X-11-175

**Published:** 2014-10-03

**Authors:** Rory H Shott, Cathy Appanah, Catherine Grenier, Guillaume Tremblay, Xavier Roucou, Luis M Schang

**Affiliations:** Department of Biochemistry and Centre for Prions and Protein Folding Diseases (CPPFD), University of Alberta, Edmonton, AB T6G 2E1 Canada; Li Ka Shing Institute of Virology, University of Alberta, Edmonton, AB Canada; Department of Biochemistry, Faculty of Medicine, Université de Sherbrooke, Sherbrooke, QC J1H 5 N4 Canada

**Keywords:** Prion disease, Cytoplasmic PrP, Neurotoxicity, Kinomics, Protein kinase, Multiplex Western blots, Akt, p70S6K, eIF4B

## Abstract

**Background:**

Dysregulated protein kinase signaling is involved in the pathogenesis of many chronic diseases. However, the dysregulated signaling pathways critical to prion pathogenesis remain incompletely characterized. Global analyses of signaling pathways may be useful to better characterize these pathways. We therefore set out to develop such global assays. To this end, we used as a model cytoplasmic mutants of the cellular prion protein (PrP^C^), which are toxic to N2a neuroblastoma cells. We tested the global assays for their sensitivity to detect changes in signaling pathways in cells expressing cytoplasmic PrP mutants.

**Methods:**

We developed a targeted proteomics (kinomics) approach using multiplex Western blots to identify signaling pathways dysregulated in chronic neurological pathologies. We tested the approach for its potential ability to detect signaling changes in N2a cells expressing cytoplasmic PrP mutants.

**Results:**

Multiplex Western blots were designed to quantitate the expression levels of 137 protein kinases in a single membrane and using only 1.2 mg of sample. The response of the blots was sensitive and linear to changes of 6% in protein levels. Hierarchical and functional clustering of the relative expression levels identified an mTOR signaling pathway as potentially dysregulated in N2a cells expressing cytoplasmic PrP. The mTOR signaling pathway regulates global protein synthesis, which is inhibited in cells expressing cytoplasmic PrP. The levels of proteins involved in the Akt1/p70S6K branch of mTOR signaling changed in synchrony with time of cytoplasmic PrP expression. Three kinases in this pathway, Akt, p70S6K, and eIF4B were in their inactive states, as evaluated by phosphorylation of their regulatory sites.

**Conclusion:**

The results presented are consistent with the previously reported inhibition of Akt/p70S6K/eIF4B signaling as mediating pathogenesis of cytoplasmic PrP. We conclude that the kinomic analyses are sensitive and specific to detect signaling pathways dysregulated in a simple *in vitro* model of PrP pathogenesis.

**Electronic supplementary material:**

The online version of this article (doi:10.1186/1743-422X-11-175) contains supplementary material, which is available to authorized users.

## Background

Dysregulation of protein kinase signaling is implicated in the pathogenesis of many chronic diseases, including neurodegenerative diseases such as Alzheimer’s and Parkinson’s [1–3]. Not surprisingly, inhibitors of protein kinases are the largest group of new cancer therapeutics [4]. Thirty-one such inhibitors are in clinical use, over 500 are involved in approximately 2,700 clinical trials, and thousands more are in various stages of pre-clinical development ([4–6] and summary of [7]). Up to 30% of the research and development budget of the pharmaceutical industry is estimated to be invested towards protein kinase inhibitors [8, 9]. Considering the importance of protein kinases in chronic disease, it would be desirable to have approaches to identify protein kinase signaling pathways that are dysregulated in chronic diseases.

Transmissible spongiform encephalopathies (TSEs), or prion diseases, are a family of chronic neurodegenerative diseases against which there are no preventative or therapeutic treatments [10]. Prion diseases are invariably lethal to humans (kuru; Creutzfeldt-Jakob disease, CJD; Gerstmann-Sträussler-Scheinker disease, GSS; fatal familial insomnia, FFI), and other species such as cattle (bovine spongiform encephalopathy, BSE), goat, sheep (scrapie), deer, elk and moose (chronic wasting disease, CWD) [11, 12]. The characteristic neuropathology of prion diseases includes gliosis, spongiform degeneration, and neuronal death. The conversion of the cellular prion protein (PrP^C^) to an abnormal conformation (PrP^Sc^) is widely accepted to be essential for pathogenesis. However, the molecular mechanisms whereby such conversion eventually mediates the consequent neurodegeneration are not yet fully understood.

Considering the critical roles that protein kinases play in the pathogenesis of other chronic neurodegenerative diseases, it is not surprising that they also participate in that of prion diseases. For example, feline Gardner-Rasheed sarcoma virus oncogene cellular homolog/Yamaguchi 73 and Esh avian sarcoma virus oncogene cellular homolog-related novel protein kinase (Fyn) knockout mice died faster than wild-type mice after scrapie infection [13]. Conversely, inhibition of protein kinase R-like endoplasmic reticulum kinase (PERK) by the overexpression of growth arrest and DNA damage-inducible protein 34 (GADD34) prolonged survival of scrapie-infected mice [14]. The activation of vascular endothelial growth factor receptor (VEGFR) inhibited death of cultured neurons treated with the neurotoxic prion peptide PrP106-126 [15]. The Abelson leukemia oncogene cellular homolog (c-Abl)/Hardy-Zuckerman 4 feline sarcoma virus oncogene cellular homolog (c-Kit)/platelet-derived growth factor receptor (PDGFR) inhibitor STI571 impaired scrapie neuroinvasion and prolonged survival of mice after intraperitoneal infection [16]. Several other inhibitors of protein kinases regulated PrP^Sc^ accumulation in scrapie-infected cultured cells [17–22]. Unfortunately, the signaling pathways most critical to prion disease pathogenesis have yet to be fully identified.

Although prion diseases are characterized by the accumulation of PrP^Sc^, endogenous PrP^C^ is also required for pathogenesis [23–26]. PrP^C^ is physiologically attached to the outside of the plasma membrane via a glycosylphosphatinositol (GPI) anchor [27, 28]. PrP^C^ may accumulate in the cytoplasm as a result of inefficient endoplasmic reticulum (ER)-targeting [29–31], ER-associated degradation [32–34], alternative translation initiation [35], or persistent pre-emptive quality control [36]. Low levels of cytoplasmic PrP have been observed in certain subpopulations of neurons without overt neurodegeneration [37–39], and the roles of cytoplasmic PrP in prion infection are disputed [40]. Nonetheless, the accumulation of PrP^C^ in the cytoplasm is often neurotoxic and has also been considered as a possible neurodegeneration mechanism [41]. Mice expressing a truncated mutant of PrP^C^ lacking its N-terminal ER-targeting and C-terminal GPI-membrane-anchoring signals (named cytoplasmic PrP, or CyPrP) suffered from ataxia with gliosis and cerebellar degeneration [42]. The molecular mechanisms of such neurodegeneration can be studied in culture because CyPrP is also toxic to mouse N2a neuroblastoma cells [42].

The expression of CyPrP inhibits heat shock protein 70 (Hsp70) synthesis in stressed N2a cells [43]. Hsp70 overexpression inhibits CyPrP-mediated toxicity, suggesting that the inhibition of Hsp70 synthesis may contribute to cell death [44, 45]. Hsp70 promotes assembly and activation of the mammalian target of rapamycin complex 2 (mTORC2; consisting of mammalian target of rapamycin [mTOR], rapamycin-insensitive companion of mTOR [rictor], mammalian lethal with SEC13 protein 8 [mLST8] and stress-activated protein kinase-interacting protein 1 [SIN1]), which then activates AKT8 virus oncogene cellular homolog (Akt)/ribosomal protein S6 kinase, 70 kilodalton, polypeptide 1 (p70S6K)/eukaryotic initiation factor 4B (eIF4B) signaling [46, 47]. Active eIF4B promotes protein synthesis, which is otherwise inhibited in cells expressing CyPrP [43, 48].

Here, we describe the development of a multiplex Western blot-based kinomics approach. Before embarking on the analysis of prion infected animals, we used a simple *in vitro* model to test the sensitivity of the approach to identify dysregulated signaling pathways, accumulation of enhanced green fluorescent protein-tagged cytoplasmic PrP (CyPrP^EGFP^) in N2a cells. The approach identified the Hsp70-regulated Akt/p70S6K/eIF4B signaling pathway to be inhibited in cells expressing CyPrP^EGFP^, consistently with previously known consequences of CyPrP^EGFP^ expression [43]. The results support the ability of the kinomics approach to detect signaling pathways dysregulated in an *in vitro* model of prion pathogenesis. As described in the companion manuscript, we have applied this approach to an *in vivo* model, infection of mice with mouse-adapted scrapie, to discover two signaling pathways dysregulated during prion disease pathogenesis.

## Results

### Multiplex Western blots quantitate the expression levels of 137 protein kinases or regulatory subunits in only 1.2 mg of sample

We developed multiplex Western blots to analyze the expression levels of protein kinases potentially involved in prion pathogenesis. In these assays, protein extracts are run in SDS-PAGE in a single well, transferred to a membrane and probed with several pools of antibodies in a multiplex Western blot apparatus.

Mice and human kinomes are well conserved, allowing the use of mice to identify and analyze protein kinases of potential importance in human disease. We performed an extensive literature search for human protein kinases that may be involved in prion or other neurodegenerative diseases (Alzheimer’s, Parkinson’s, Huntington’s, multiple sclerosis, or amyotrophic lateral sclerosis). We also included protein kinases involved in cellular pathologies associated with prion disease (neuronal apoptosis, gliosis, glial activation, neuronal degeneration, or neuronal survival). The search was restricted to protein kinases the mouse orthologs of which were detected by antibodies commercially available at the time. Following these criteria, we selected 145 protein kinases, almost 30% of the 540 or 518 protein kinases in the mouse or human kinomes, respectively (Additional file 1: Figure S1) [49, 50]. The selected protein kinases are distributed among the eight groups of protein kinases (AGC, CAMK, CMGC, CK1, STE, TK, TKL, and atypical) [50]. The most under-represented kinases in the selection are involved in muscle contraction [myosin light chain kinases, MLCK], spermatogenesis [testis specific serine/threonine kinases, TSSK], or developmental processes [transforming growth factor-beta receptor kinases, TGF-β]), which are not expected to be critical in prion disease. We also included the eleven cyclins or cyclin-like proteins (p25/p35, p39), which are the activating subunits required for the activity of the catalytic cyclin-dependent kinase (CDK) moiety of the active CDK/cyclin heterodimers.

Our long-term objective was to analyze the kinomic changes in brains of scrapie-infected mice (see companion paper). We therefore optimized the antibodies in multiplex Western blots with mouse brain homogenate. Antibodies specific for 122 protein kinases or regulatory subunits (Additional file 2: Table S1) recognized their cognate proteins in 1.2 mg of mouse brain homogenate (200 μg loaded per linear cm). We selected the dilution of each antibody that resulted in maximum signal intensity and minimum background with no antibody saturation (i.e., signal did not increase with increasing antibody concentrations). Antibodies specific for calcium/calmodulin-dependent kinase 4 (CaMK4), mitogen-activated protein kinase/extracellular signal-regulated kinase 5 (MEK5), and Jun N-terminal kinase 2 (JNK2) detected two isoforms each. Fifteen antibodies specific for proteins not recognized in mouse brain homogenate were optimized in Western blots using lysate from cultured 3T3 mouse fibroblasts (200 μg loaded per linear cm). The remaining 19 antibodies did not detect their cognate protein in mouse brain homogenate or 3T3 cell lysates.

The multiplex Western blots were tested for reproducibility. Mouse brain homogenate resolved throughout a single-well gel (1.2 mg; 200 μg per linear cm) was transferred, and 16 lanes were isolated in the membrane with a multi-screen apparatus. The extracellular signal-regulated kinases (Erk) 1 and 2 were probed in each of the 16 lanes. The standard deviation between all 16 lanes was only 2.2% or 1.0% of the average for Erk1 or Erk2, respectively, and the range was 8% of the average for Erk1 or 4% of Erk2.

**Figure 1:**
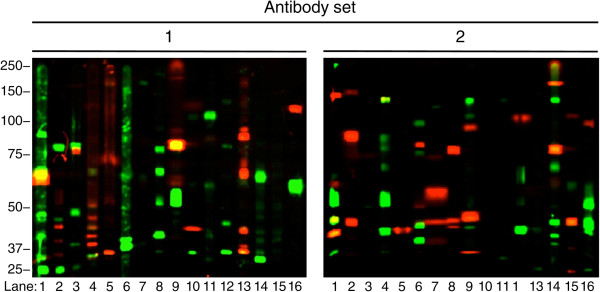
**Multiplex Western blots detect 122 selected protein kinases in only 1.2 mg of mouse brain.** A single-well gel was loaded with 1.2 mg of mouse brain homogenate, and the proteins were resolved and transferred. A multi-screen apparatus isolated 16 individual lanes within the area of homogeneously resolved protein and probed with optimized antibodies specific for 122 selected protein kinases. Molecular weights in kDa are indicated on the left. Signal from secondary antibody labeled with Alexa Fluor 680 (red bands) and IRDye 800 (green bands) was detected using a LI-COR Odyssey infrared imaging system. Yellow bands, red-labeled goat anti-mouse secondary antibody detected by green-labeled anti-goat secondary antibody. Due to the wide range in expression levels, no single exposure of the blot can show all the bands. The bands that are visible in each lane at the exposure shown are listed (from top to bottom) in the main text.

To minimize the variability and amount of sample required, multiple proteins were probed for in each lane of a single membrane. The 137 proteins were grouped into 32 sets such that each set contained proteins of molecular weights clearly resolved in SDS-PAGE, detected by antibodies of different species and recognized by the antibodies giving the weakest or strongest signals (Sets 1 and 2, respectively). The membranes were probed first for the 16 sets containing the proteins that resulted in the lowest signal intensities (Figure 1), stripped (only once) and reprobed for the remaining 16 sets. All 122 protein kinases or regulatory subunits previously detected in standard Western blots were detected in the multiplex blots. The following protein kinases were detected in Set 1. **Lane 1:** DAPK1 (not visible, 145 kDa), non-specific (green, 95 kDa), Syk (not visible, 74 kDa), CaMK4β (not visible, 66 kDa), CaMK4 (red, 63 kDa), CK1γ1 (green, 45 kDa), non-specific (green, 42 kDa), cyclin D3 (red, 33 kDa), non-specific (green, 32, 25 kDa). DAPK1, Syk, and CaMK4β are not visible at the exposure shown. **Lane 2:** HER2 (not visible, 160 kDa), RSK1 (green, 85 kDa), AMPKα1 (not visible, 64 kDa), CK1γ2 (not visible, 55 kDa), non-specific (green, 45 kDa; red, 43, 40, 35 kDa; green, 25 kDa). HER2, AMPKα1, and CK1γ2 are not visible at the exposure shown. **Lane 3:** ROCK1 (not visible, 162 kDa), GRK2 (green, 80 kDa), p70S6K (red, 75 kDa), PCTAIRE3 (green, 48 kDa), non-specific (green, 40 kDa), cyclin H (not visible, 37 kDa), non-specific (green, 27 kDa). ROCK1 and cyclin H are not visible at the exposure shown. **Lane 4:** JAK1 (red, 125 kDa), MARK4 (not visible, 80 kDa), PLK1 (red, 66 kDa), non-specific (green, 65 kDa; red, 52 kDa), MAPKAPK2 (not visible, 48 kDa), non-specific (red, 48, 45, 42, 40 kDa), p25/p35 (green, 35 kDa). MARK4 and MAPKAPK2 are not visible at the exposure shown. **Lane 5:** HER3 (red, 185 kDa), Raf1 (red, 71 kDa), Fms/CSF1R (not visible, 50 kDa), cyclin D1 (red, 36 kDa). Fms/CSF1R is not visible at the exposure shown. **Lane 6:** CRIK (not visible, 220 kDa), MSK1 (not visible, 90 kDa), non-specific band (green, 65, 45, 38 kDa). CRIK and MSK1 are not visible at the exposure shown. **Lane 7:** Non-specific (green, 170 kDa), MLK3 (not visible, 90 kDa), PDK1 (green, 60 kDa), CK1α (not visible, 42 kDa), CK2α1 (not visible, 40 kDa). MLK3, CK1α, and CK2α1 are not visible at the exposure shown. **Lane 8:** Non-specific (green, 80 kDa), GRK5 (green, 65 kDa), non-specific (green, 52 kDa), p38α (not visible, 42 kDa), non-specific (green, 40 kDa). p38α is not visible at the exposure shown. **Lane 9:** Non-specific (red, 250 kDa), PKD2 (not visible, 98 kDa), PKCβ (red, 82 kDa), non-specific (red, 60 kDa), DLK (green, 51 kDa), CDK7 (not visible, 41 kDa). PKD2 and CDK7 are not visible at the exposure shown. **Lane 10:** TrkB (red, 130 kDa), Erk5 (green, 110 kDa), MST1 (not visible, 60 kDa), CK1ϵ (red, 44 kDa), non-specific (green, 34 kDa). MST1 is not visible at the exposure shown. **Lane 11:** PKD1 (green, 112 kDa), IKKβ (not visible, 88 kDa), Akt3 (green, 60 kDa), MKK7 (not visible, 46 kDa), p38β (green, 42 kDa). IKKβ and MKK7 are not visible at the exposure shown. **Lane 12:** EphA1 (not visible, 180 kDa), InsR (not visible, 130 and 88 kDa), non-specific (green, 80 kDa), RIPK2 (not visible, 60 kDa), non-specific (green, 45, 35 kDa). EphA1, InsR, and RIPK2 are not visible at the exposure shown. **Lane 13:** ATM (not visible, ~300 kDa), PRK2 (not visible, 130 kDa) B-Raf (red, 90 kDa), non-specific (red, 65 kDa), Myt1 (green, 63 kDa), non-specific (red, 42, 35 kDa). ATM and PRK2 are not visible at the exposure shown. **Lane 14:** c-Abl (not visible, 130 kDa), PAK3 (green, 65 kDa), CaMK1α (green, 42 kDa). c-Abl is not visible at the exposure shown. **Lane 15:** PKD3 (not visible, 95 kDa), cyclin A (not visible, 60 kDa), both are not visible at the exposure shown. **Lane 16:** Non-specific (red, 120 kDa), Akt2 (green, 60 kDa), Lck (not visible, 56 kDa). Lck is not visible at the exposure shown. The following protein kinases were detected in Set 2. **Lane 1:** ROCK2 (red, 183 kDa), Akt1 (red, 60 kDa), SGK3 (green, 50 kDa), non-specific (green, 45 kDa), Erk2 (red, 42 kDa), non-specific (green, 36 kDa). **Lane 2:** TNIK (red, 180 kDa), PKCϵ (red, 90 kDa), MEK2 (red, 45 kDa). **Lane 3:** EphA7 (not visible, 86 kDa), PKCδ (not visible, 78 kDa), GSK3α (not visible, 50 kDa), all are not visible at the exposure shown. **Lane 4:** Non-specific (green, 140 kDa), DYRK1A (green, 90 kDa), IKKα (not visible, 80 kDa), non-specific (green, 80 kDa), PKR (not visible, 66 kDa), non-specific (green, 63 kDa), PKACβ (green, 53 kDa), non-specific (green, 42 kDa). IKKα and PKR are not visible at the exposure shown. **Lane 5:** TrkC (not visible, 145 kDa), CaMK2γ (not visible, 60 kDa), cyclin E1 (not visible, 55 kDa), PKACα (red, 42 kDa). TrkC, CaMK2γ, and cyclin E1 are not visible at the exposure shown. **Lane 6:** DDR1 (green, 109 kDa), PKCζ (red, 86 kDa), non-specific (green, 80 kDa), JNK2α2/β2 (red-partly covered by adjacent non-specific band, 52 kDa), non-specific (green, 44 kDa), JNK2α1/β1 (red, 42 kDa), MKK6 (green, 38 kDa), cyclin G1 (not visible, 29 kDa). cyclin G1 is not visible at the exposure shown, and JNK2α1/β1 due to signal from adjacent non-specific band. **Lane 7:** TrkA (not visible, 138 kDa), PKCι (not visible, 75 kDa), MEK5α (red, 55 kDa), MEK5β (red, 45 kDa). TrkA and PKCι are not visible at the exposure shown. **Lane 8:** PKCγ (red, 80 kDa), CaMK1δ (not visible, 44 kDa), non-specific (red, 45, 43 kDa). CaMK1δ is not visible at the exposure shown. **Lane 9:** EphA3 (green, 140 kDa), non-specific (green, 120 kDa), CASK (red, 104 kDa), GSK3β (red, 46 kDa), p38γ (green, 43 kDa), CDK5 (red, 30 kDa). **Lane 10:** ASK1 (not visible, 155 kDa) and p39 (not visible, 40 kDa), both are not visible at the exposure shown. **Lane 11:** MEKK1 (not visible, 205 kDa), PINK1 (not visible, 66 kDa), Fyn (not visible, 59 kDa), and p38δ (not visible, 43 kDa), all are not visible at the exposure shown. **Lane 12:** EphA4 (red, 120 kDa), PAK1 (green, 68 kDa), Lkb1 (not visible, 55 kDa), PKACγ (green, 40 kDa). Lkb1 is not visible at the exposure shown. **Lane 13:** HER4 (not visible, 182 kDa), JAK2 (not visible, 122 kDa), PKG1 (not visible, 76 kDa), Src (not visible, 60 kDa), Nek6 (green, 45 kDa). HER4, JAK2, PKG1, and Src are not visible at the exposure shown. **Lane 14:** SLK (red, 220 kDa), HGK (green, 140 kDa), PKCα (red, 82 kDa), LIMK1 (green, 70 kDa), Erk1 (green, 44 kDa), non-specific (green, 40, 38 kDa). **Lane 15:** PRK1 (red, 120 kDa), CaMK2β (green, 66 kDa), MEK1 (red, 45 kDa), CDKL1 (not visible, 42 kDa). CDKL1 is not visible at the exposure shown. **Lane 16:** Pyk2 (red, 115 kDa), CaMKK2 (not visible, 66 kDa), JNK1 (green, 50 kDa), non-specific (green, 45 kDa). CaMKK2 is not visible at the exposure shown.

Importantly, the multiplex Western blots allowed for the identification and resolution of: *(i)* kinases yielding high and low intensity signal (for example, Set 1; Akt3 in lane 11 [green band at 60 kDa] vs. Akt2 in lane 16 [green band at 60 kDa]); *(ii)* kinases of similar molecular weight in the same lanes (for example, Set 1, lane 3; GRK2 [green band at 80 kDa] and p70S6K [red band at 75 kDa]); and *(iii)* as many as 5 protein kinases in a single lane (for example, Set 2 lane 9: EphA3 [green band at 140 kDa], CASK [red band at 104 kDa], GSK3β [red band at 46 kDa], p38γ [green band at 43 kDa], CDK5 [red band at 30 kDa]; Set 2, lane 14; SLK [red band at 220 kDa], HGK [green band at 140 kDa], PKCα [red band at 82 kDa], LIMK1 [green band at 70 kDa], Erk1 [green band at 44 kDa]) (Figure 1).

In summary, we developed multiplex Western blots to quantitate 137 protein kinases or regulatory subunits involved in neurological diseases or pathologies using only 1.2 mg of sample on a single membrane stripped only once.

### Multiplex Western blots are sensitive and linear, detecting incremental 6 (or 3)% changes in protein levels

**Figure 2:**
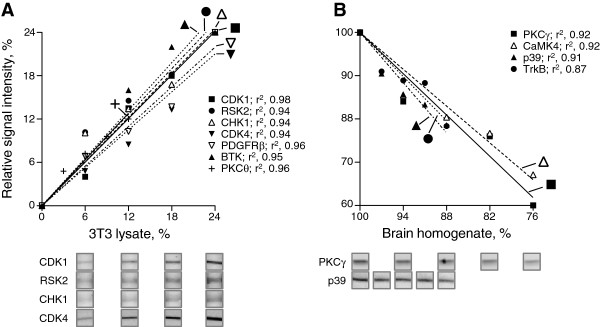
**Multiplex Western blots are sensitive and linear, detecting incremental 6 (or 3)% changes in protein levels.** Line graphs presenting the relative signal intensity of the indicated protein kinases expressed in 3T3 cells but not in brain **(A)**, plotted against the percentage of 3T3 lysate, or the protein kinases expressed in brain but not in 3T3 cells **(B)**, plotted against the percentage of mouse brain homogenate. Mouse brain homogenate was spiked with incremental 3, 6, 9, or 12%, or 6, 12, 18, or 24% 3T3 cell lysate. The resolved proteins were transferred and probed by multiplex Western blot. The Western blots for CDK1, RSK2, CHK1, CDK4 **(A)**, PKCγ **(B)** at 6% incremental changes, and p39 **(B)** at 3% incremental changes, are shown at the bottom of each graph as examples. The regression coefficient (r^2^) for each protein is indicated.

We next tested the variability and linearity of the multiplex Western blots to increases in protein levels. Fifteen of the 137 protein kinases or regulatory subunits were not detected in mouse brain homogenate but were detected in cell lysate from 3T3 mouse fibroblasts. Mouse brain homogenate was spiked with incremental 6% (average of the range of the reproducibility of quantitations, as evaluated for Erk1 and Erk2) increases of 3T3 cell lysate, from 0 to 24%. Six of the proteins detected only in 3T3 cell lysates (CDK1, CDK4, PDGFRβ, ribosomal protein S6 kinase 2 [RSK2], checkpoint kinase 1 [CHK1], Bruton’s tyrosine kinase [BTK]) were quantitated. Their levels increased linearly (r^2^ ≥ 0.94, *P* < 0.001) along the increases in 3T3 cell lysate from 0 to 24% (Figure 2A), and those of protein kinase C theta (PKCθ) with incremental 3% increases of the 3T3 cell lysate from 0 to 12% (r^2^ = 0.96, *P* < 0.0001). To test the sensitivity to decreases in protein levels, we analyzed the levels of four protein kinases (PKCγ, CaMK4, p39, and tropomyosin-related kinase B [TrkB]) detected in mouse brain but which are not expressed in 3T3 cells [51–55]. Their levels decreased linearly (r^2^ ≥ 0.87, *P* < 0.01) along the incremental 6% (PKCγ, CaMK4) or 3% (TrkB, p39) decreases of mouse brain homogenate from 100 to 76% (Figure 2B). The multiplex Western blots are reproducible, sensitive and linear, detecting incremental 6% increases or decreases in protein levels.

### Primary kinomic screens of N2a cells expressing cytoplasmic PrP mutants identified the mTOR signaling pathway as potentially dysregulated

**Figure 3:**
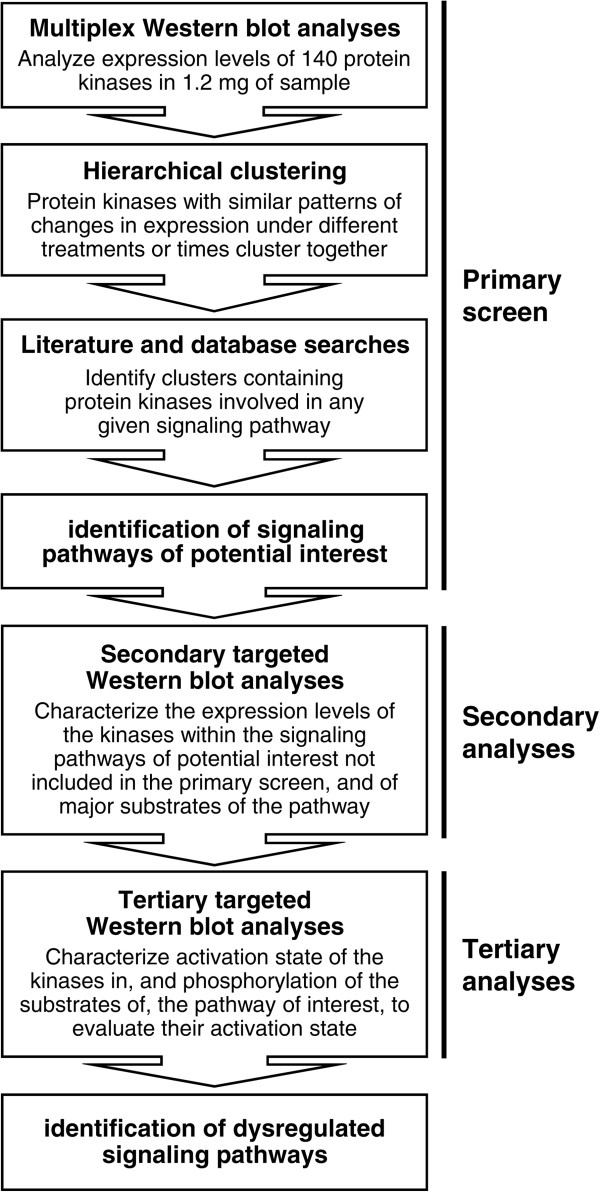
**Flow chart of the kinomic analyses.** Algorithm used to identify dysregulated signaling pathways.

To identify potentially dysregulated signaling pathways, protein kinases with similar changes in relative expression levels were blindly clustered by agglomerative unsupervised hierarchical clustering (Figure 3). Any clusters containing protein kinases involved in any given signaling pathways were next identified by literature and signal transduction database searches. The different treatments used in the clustering must affect the same signaling pathways differently, or affect different signaling pathways altogether, for this approach to detect relevant clusters.

The neurotoxicity of CyPrP expression requires residues 116–156 [44]. We transfected N2a neuroblastoma cells with CyPrP or two mutants truncated within this region, CyPrP124stop and CyPrP124-230. The expression of CyPrP124stop is not toxic to N2a cells [56], and cells expressing CyPrP124-230 also are healthy, although toxicity has not been quantitatively assessed. We expected cells expressing CyPrP to affect different subsets of signaling pathways than, or to differentially affect the same signaling pathways as, those expressing CyPrP124stop or CyPrP124-230. Such differentially affected signaling pathways might be involved in CyPrP-mediated neurotoxicity.

**Figure 4:**
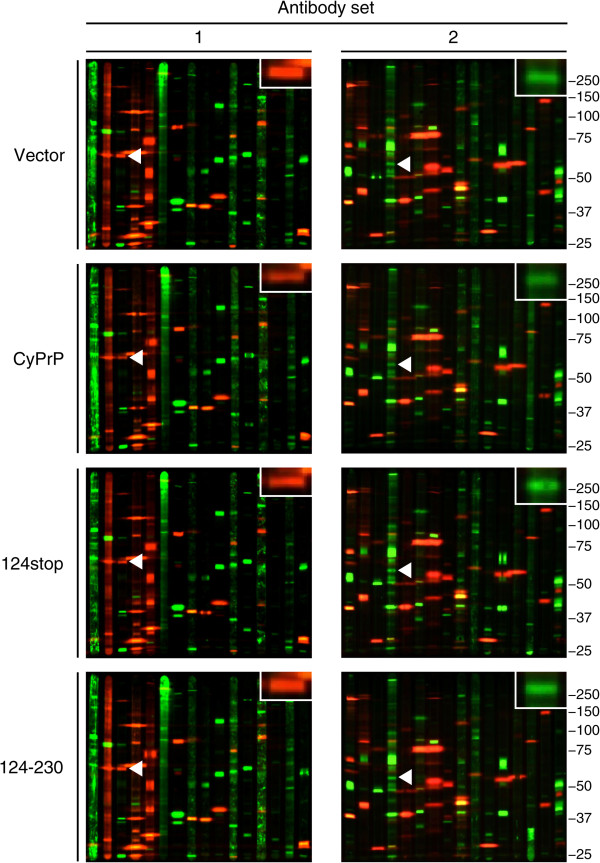
**Differential expression of protein kinases in N2a cells expressing different cytoplasmic PrP mutants.** Multiplex Western blots of lysates from N2a cells transfected with empty vector (vector), or vector encoding CyPrP^EGFP^ (CyPrP), CyPrP^EGFP^124stop (124stop), or CyPrP^EGFP^124-230 (124–230). Molecular weights in kDa are indicated on the right. The protein kinases p70S6K (set 1) and PKACβ (set 2) are indicated by white arrowheads and enlarged, to illustrate their differential expression in cells expressing the different mutants.

The cytoplasmic PrP mutants were tagged with enhanced green fluorescent protein (EGFP) to evaluate transfection efficiencies (Additional file 3: Figure S2), as described previously [56]. Lysates collected from N2a cells 24 h after transfection with empty vector, used as control, or vector encoding for CyPrP^EGFP^, CyPrP^EGFP^124stop, or CyPrP^EGFP^124-230 were subjected to multiplex Western blot (Figure 4, Additional file 4: Figure S3). The densitometric data from the 76 protein kinases detected in cells expressing all mutants were normalized to the levels in the cells transfected with the empty vector and then analyzed by unsupervised hierarchical clustering.

We first performed hierarchical clustering of the three PrP mutants to evaluate any potential (unexpected) similarities in changes in protein kinase expression. If two different cytoplasmic PrP mutants resulted in identical changes in protein kinase expression, the correlation would be 1; a correlation of 0 indicates no relationship. The city-block distance metric correlation between CyPrP^EGFP^124stop and CyPrP^EGFP^124-230 was 0.088, and between them and CyPrP^EGFP^ was 0. The lack of the correlation indicated that, as expected, the different cytoplasmic PrP mutants dysregulated different signaling pathways.

**Figure 5:**
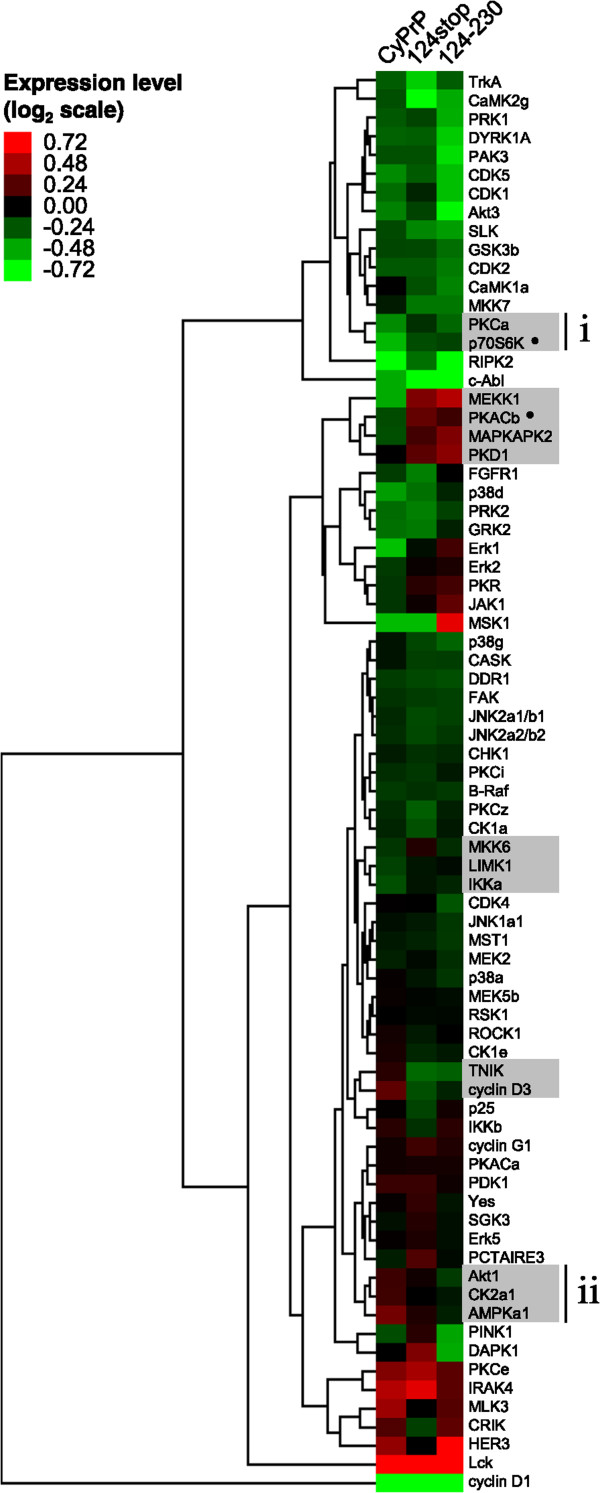
**Identification of the mTOR signaling pathway as potentially dysregulated in cells expressing CyPrP** ^**EGFP**^ **.** Hierarchical clustering of 76 protein kinases using the normalized and log_2_ transformed densitometric data from primary multiplex Western blots. Red, higher expression levels; green, lower expression levels. Each category encompasses changes in expression levels of 18% (0.23 in log_2_ scale), 3 times the 6% linear changes detected by the technique. Grey boxes indicate clusters of protein kinases most differentially expressed in cells expressing CyPrP^EGFP^. The clusters **(** ***i*** **)** and **(** ***ii*** **)** consist of PKCα, p70S6K, Akt1, and AMPKα1 involved in mTOR signaling. The protein kinases highlighted in Figure 4, p70S6K and PKACβ, are indicated by (●).

We next performed hierarchical clustering of protein kinases by their expression levels in cells transfected with each of the three cytoplasmic PrP mutants (Figure 5). The log_2_ relative expression levels were classified in categories each encompassing 18% changes in expression, three times the 6% changes that the tests detect linearly (Figure 2). We then identified the clusters that contained protein kinases involved in any given signaling pathways. We were most interested in clusters containing protein kinases that were expressed to different levels in cells expressing CyPrP^EGFP^ than in cells expressing CyPrP^EGFP^124stop or CyPrP^EGFP^124-230. We excluded clusters containing protein kinases expressed to similar levels in cells expressing empty vector or CyPrP^EGFP^. Five clusters were identified following these criteria (Figure 5, grey boxes). The mTOR signaling pathway includes the mTOR complex 2 (mTORC2), which activates Akt, PKCα, and SGK1, and the mTOR complex 1 (mTORC1), which activates p70S6K and eukaryotic initiation factor 4E-binding protein (eIF4E-BP) [57]. Two clusters containing protein kinases most affected in cells expressing CyPrP^EGFP^ included all the mTORC substrates included in the primary screens (PKCα, Akt, p70S6K) (Figure 5, ***i*** and ***ii***). PKCα and p70S6K clustered together because their levels were lowest in cells expressing CyPrP^EGFP^. Adenosine monophosphate-activated protein kinase catalytic subunit alpha-1 (AMPKα1), which also regulates mTOR signaling, clustered together with Akt1 because their levels were highest in cells expressing CyPrP^EGFP^. The mTOR signaling pathway regulates protein synthesis, which is inhibited in cells expressing CyPrP^EGFP^[43]. The results from the primary kinomic screens therefore suggested that CyPrP-mediated neurotoxicity in N2a cells might involve dysregulated mTOR signaling.

### The levels of proteins in the Akt1/p70S6K branch of the mTOR signaling pathway decreased synchronously with time of CyPrP^EGFP^ expression

If the changes in the levels of the proteins involved in mTOR signaling were the result of CyPrP^EGFP^ expression, then their levels would be expected to change in synchrony with time of expression. We analyzed three sets of N2a cell lysates prepared 12, 24 and 48 h after transfection with the CyPrP^EGFP^-expressing construct in three independent biological repeats. Cells expressing EGFP were used as control.

**Figure 6:**
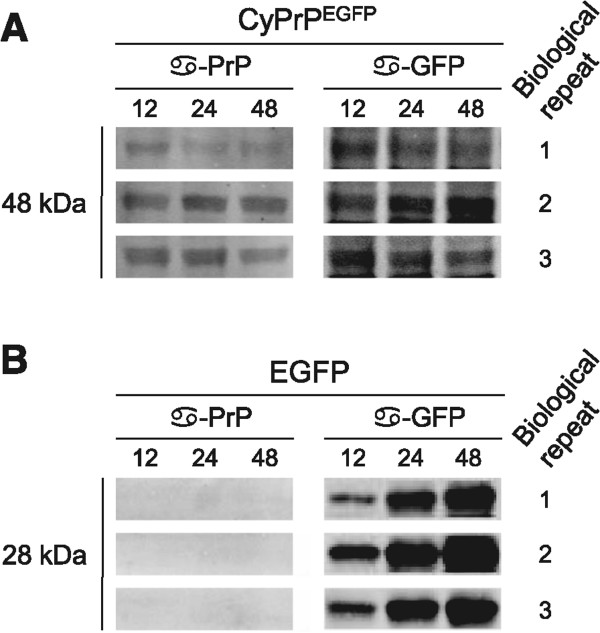
**Levels of CyPrP** ^**EGFP**^ **and EGFP in the samples used for targeted secondary and tertiary analyses.** Western blots of lysates from three biological repeats expressing CyPrP^EGFP^ **(A)** or EGFP **(B)** for 12, 24, and 48 h. CyPrP^EGFP^ (48 kDa) was detected by α-PrP and α-GFP primary antibodies, and EGFP (28 kDa) by α-GFP antibody only. Different exposures are shown for **(A)** and **(B)**.

The levels of EGFP increased from 12 to 48 h after transfection (Figure 6). In contrast, those of CyPrP^EGFP^ changed little (slightly decreased) with time. Targeted secondary analyses characterized the expression levels of ten proteins involved in the mTOR signaling pathway. Four of the antibodies used in these targeted multiplex Western blots had already been used in the primary screen (Akt1, p70S6K, PKCα, AMPKα1). New antibodies were selected to analyze two protein kinases (mTOR and mitogen-activated protein kinase-interacting kinase 1 [Mnk1]) and four downstream substrates (eIF4B, eukaryotic initiation factor 4E [eIF4E], ribosomal protein S6 [S6], and eukaryotic elongation factor 2 [eEF2]), which were not included in the primary screens. These antibodies were optimized as those used in the primary screens. The normalized expression levels were grouped into categories spanning 20% changes, slightly above 3 times the 6% changes that the tests detect linearly (Figure 2).

**Figure 7:**
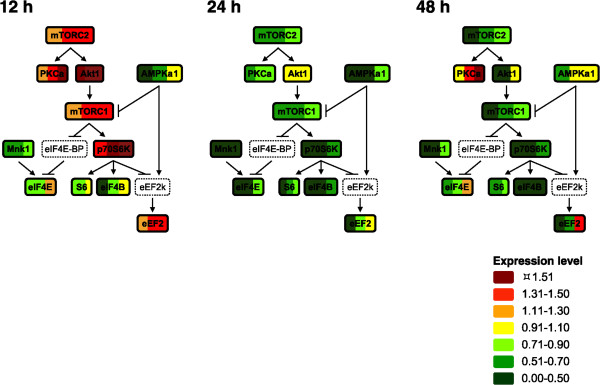
**Lower levels of mTOR signaling proteins in cells expressing CyPrP** ^**EGFP**^ **for 24 and 48 h.** Targeted secondary analyses of mTOR signaling in N2a cells expressing CyPrP^EGFP^ for 12, 24, or 48 h. The normalized expression levels of 10 protein kinases or substrates in each of the three biological repeats are shown by individual color bars. Each color-coded category encompasses 20% changes in the levels of expression, greater than 3 times the 6% linear changes detected by the technique. Proteins indicated by dashed lines were not analyzed. The expression levels of mTORC1 and mTORC2 represent the levels of mTOR.

**Figure 8:**
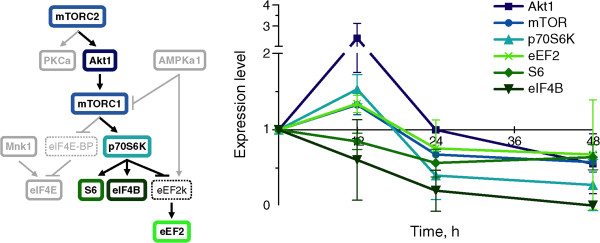
**The levels of proteins in the Akt1/p70S6K branch of the mTOR signaling pathway change coordinately.** Time-course analyses of the normalized expression levels of Akt1, mTOR, p70S6K, S6, eIF4B, and eEF2 in N2a cells expressing CyPrP^EGFP^ for 12, 24, or 48 h. Mean ± SD; *n* = 3.

The levels of Akt1, mTOR, p70S6K, eEF2, and PKCα were higher at 12 h in all three samples of cells expressing CyPrP^EGFP^ than in those of cells expressing EGFP (Figure 7). At 24 h, in contrast, the levels of all proteins analyzed were consistently lower than (or at the most, equal to) those in cells expressing EGFP. Little change was observed from 24 to 48 h, with exception to PKCα, eIF4E, and eEF2, which were expressed to their highest levels in the cells expressing the lowest levels of CyPrP^EGFP^. We performed a time-course analysis of the proteins in the Akt1/p70S6K branch of the mTOR signaling pathway (Figure 8). Akt1, mTOR, p70S6K, and eEF2, in the Akt1/p70S6K branch, were expressed to higher levels in cells expressing CyPrP^EGFP^ than in cells expressing EGFP at 12 h, and then to lower levels at 24 or 48 h (except for Akt at 24 h). The levels of the other p70S6K substrates tested, S6 and eIF4B, also decreased with time. In summary, the levels of the proteins involved in Akt1/p70S6K signaling decreased synchronously after 12 h of CyPrP^EGFP^ expression.

### Inhibition of Hsp70-regulated Akt/p70S6K/eIF4B signaling in cells expressing CyPrP^EGFP^

To test whether Akt1/p70S6K signaling was dysregulated in cells expressing CyPrP^EGFP^, we characterized the activation states of the ten proteins previously tested. We optimized phosphorylation-specific antibodies for the sites directly phosphorylated by the relevant upstream protein kinases. Akt is activated by phosphorylation on S473 by mTORC2 and on T308 by 3-phosphoinositide-dependent protein kinase 1 (PDK1) [58, 59]. The synthesis of Hsp70, which activates mTORC2, is inhibited in cells expressing CyPrP [43, 46]. We focused on Hsp70-regulated phosphorylation of Akt1. No available antibody was specific for the phosphorylation at the activation-specific site S473 on Akt1 only. We used an antibody that detects S473 phosphorylation on all Akt isoforms (Akt1, 2, and 3). Although mTOR phosphorylation is not required for mTORC1 activation, active mTORC1 typically contains S2448 phosphorylated mTOR [60]. We included an antibody specific for this phosphorylation (P-S2448). We also included antibodies specific for the phosphorylation at activation-specific sites (activating phosphorylation) on AMPKα (P-T172), Mnk1 (P-T197/202), PKCα (P-S657), p70S6K (P-T389), S6 (P-S235/236; P-S240/244), eIF4B (P-S422) and eIF4E (P-S209), or the inhibition-specific site (inhibitory phosphorylation) on eEF2 (P-T56) [61–67]. The phosphorylation level of Akt (P-S473) at 12 h could be tested in only two of the three independent biological replicates due to limiting sample.

**Figure 9:**
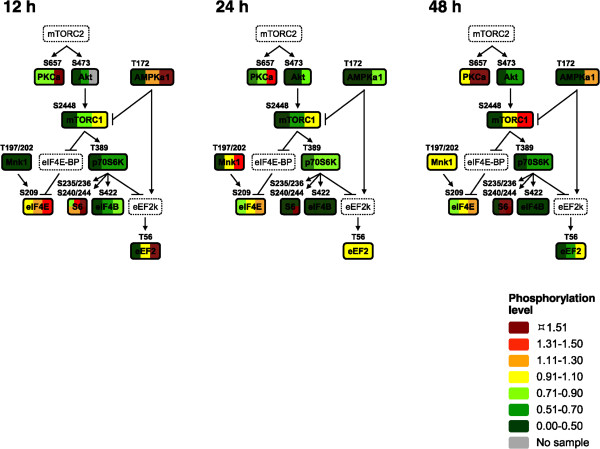
**Lower levels of activating phosphorylation of Akt, p70S6K, and eIF4B in cells expressing CyPrP** ^**EGFP**^ **.** Targeted tertiary analyses of mTOR signaling in N2a cells expressing CyPrP^EGFP^ for 12, 24, or 48 h. The normalized absolute phosphorylation levels of 10 protein kinases or substrates in each of the three biological repeats are shown by the color bars. The phosphorylation sites evaluated are indicated above each protein. Proteins indicated by dashed lines were not analyzed. Due to limited sample, the levels of p-Akt at 12 h were measured only in two of the three biological repeats. The color bars for S6 indicate the normalized phosphorylation levels of S235/236 (top) and S240/244 (bottom).

**Figure 10:**
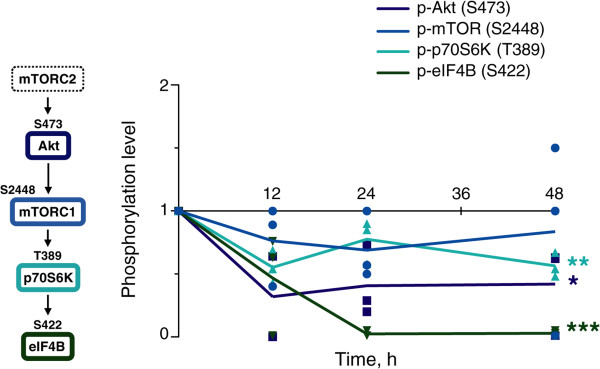
**Inhibition of the Akt/p70S6K/eIF4B signaling pathway in cells expressing CyPrP** ^**EGFP**^ **.** Time-course analysis of the normalized absolute levels of phosphorylated Akt (P-S473), mTOR (P-S2448), p70S6K (P-T389), and eIF4B (P-S422) in N2a cells expressing CyPrP^EGFP^ for 12, 24, or 48 h; individual data and mean (p-Akt, square; p-mTOR, circle; p-p70S6K, triangle; p-eIF4B, inverted triangle). Due to limited sample, the levels of p-Akt at 12 h were only measured in two biological repeats. The levels of all others were evaluated in three independent biological repeats. Differences in changes in phosphorylation levels in cells expressing CyPrP^EGFP^ and EGFP was analyzed by replicates test for lack-of-fit. *, *P* < 0.05; **, *P* < 0.01; ***, *P* < 0.001.

Active mTORC2 activates Akt by phosphorylation on S473, which then activates mTORC1 which, in turn, activates p70S6K by phosphorylation on T389. Active p70S6K activates eIF4B by phosphorylation on S422. The levels of activated Akt (P-S473), p70S6K (P-T389), and eIF4B (P-S422) were consistently lower in cells expressing CyPrP^EGFP^ than in cells expressing EGFP at all times (Figure 9). Phosphorylated mTOR (P-S2448) levels were also lower, or equal, in cells expressing CyPrP^EGFP^ than in those expressing EGFP, except for one sample at 48 h. We performed nonlinear regression analyses (the regressions are non-linear) to test whether the changing phosphorylation levels of any proteins involved in the mTOR signaling pathway were different in cells expressing CyPrP^EGFP^ or EGFP. The levels of activated Akt (P-S473), p70S6K (P-T389), and eIF4B (P-S422) were different in cells expressing CyPrP^EGFP^ or EGFP (replicates test for lack-of-fit; Akt [P-S473], *P* = 0.02; p70S6K [P-T389], *P* = 0.001; eIF4B [P-S422], *P* = 0.0002) (Figure 10). In conclusion, Akt/p70S6K/eIF4B signaling is inhibited in cells expressing CyPrP^EGFP^.

## Discussion

Here we describe the development of kinomic analyses aimed at identifying signaling pathways dysregulated during chronic pathologies. We designed and optimized multiplex Western blots to quantitate the expression of 137 protein kinases (including regulatory subunits) in a single membrane, and using only 1.2 mg of sample. These multiplex Western blots were reproducible, sensitive and linear, detecting 6% incremental changes in protein level. We tested the multiplex Western blots in a kinomic screen of an *in vitro* model of prion pathogenesis, N2a neuroblastoma cells expressing cytoplasmic PrP mutants. The mTOR signaling pathway was identified in the primary screen. The levels of proteins involved in the Akt1/p70S6K branch of the mTOR signaling pathway changed synchronously and were phosphorylated or unphosphorylated to their inhibited states in CyPrP^EGFP^ expressing cells.

Hsp70 overexpression inhibits CyPrP-mediated toxicity and the synthesis of Hsp70 is inhibited in cells expressing CyPrP^EGFP^[43–45]. The inhibition of Hsp70-regulated Akt/p70S6K/eIF4B signaling in cells expressing CyPrP^EGFP^ is fully consistent with those previous data [43–45], supporting the ability of the approach to detect kinomic changes. Inhibition of Hsp70-activated Akt/p70S6K/eIF4B signaling may also be important in CyPrP pathogenesis. Depletion of eIF4B by RNA interference promotes cell death [48]. Hsp70 overexpression protects against this cell death in part by promoting the expression of the anti-apoptotic protein B-cell CLL/lymphoma 2 (Bcl-2) [68, 69]. Active eIF4B is also required for the translation of Bcl-2 (and other proteins translated from mRNAs with highly structured 5’ untranslated regions). CyPrP may promote cell death by inhibiting Bcl-2 synthesis through the Akt/p70S6K/eIF4B pathway. Overexpression of Akt1, mTOR, and p70S6K in cells expressing CyPrP^EGFP^ for 12 h may well be an ultimately fruitless early attempt to overcome neurotoxic inhibition of Akt/p70S6K/eIF4B signaling.

Active eIF4B promotes translation initiation by stimulating the helicase activity of eukaryotic initiation factor 4A (eIF4A) and promoting ribosome binding [70–73]. Global protein synthesis is inhibited in cells expressing CyPrP^EGFP^[43]. Previous studies have indicated that eukaryotic initiation factor 2 alpha (eIF2α) was inhibited in cells expressing CyPrP^EGFP^ in a protein kinase R (PKR)-dependent manner [43]. The inhibition of eIF4B also inhibits protein synthesis [48], suggesting that the inhibition of Akt/p70S6K/eIF4B signaling may too contribute to the inhibition of protein synthesis in cells expressing CyPrP^EGFP^.

The kinomics approach we describe here is dependent on the primary screens to first identify signaling pathways of potential interest. The protein kinases for the primary screens were selected based on their potential roles in prion or other neurodegenerative diseases, or in pathologies associated with prion disease. However, the screens are extensive, evaluating almost one-third of the protein kinases in the mouse (or human) genome. Other kinomic analyses [15] used peptide arrays to identify different signaling pathways induced by PrP^C^ stimulation with a PrP antibody (6H4) or a small peptide (PrP106-126). These assays are limited by the availability of the peptide arrays and the different activities of several kinases on small peptide or protein substrates. Other previous studies have only analyzed a limited number of protein kinases (c-Abl, Src, Fyn, Yes, Lck, Lyn, Syk, Akt, mTOR, p70S6K, CaMK2α, CDK5, PYK2, PKA, PKC, PKR, PERK, MEK1/2 and MAPKs), based mostly on their hypothesized involvement in prion disease pathogenesis [13, 14, 17–20, 22, 74–88]. Such analyses provide information about the selected signaling pathways only. High-throughput reverse-phase protein arrays [89] can also be used to analyze changes in protein kinase levels. This approach requires highly specific antibodies, however, limiting the number of protein kinases that can be analyzed. The antibodies included in the experiments described here are also specific. Nonetheless, multiplex Western blots discriminate specific from non-specific binding (by molecular weight), tolerating some level of cross-reactivity. More proteins can therefore be analyzed by the multiplex Western blot approach than by protein arrays.

High-throughput gene arrays have identified hundreds of genes expressed differentially during, and a number of cellular processes affected by, prion diseases [90–102]. However, only 518 or 540 of the approximately 30,000 genes in the human or mouse genomes encode for protein kinases. Hierarchical clustering of gene array data thus results in very few clusters with multiple protein kinases, making it difficult to identify any particular signaling pathway. Moreover, protein kinases are extensively regulated post-transcriptionally and therefore their mRNA and protein levels often do not correspond [103]. Global analyses of changes in microRNA (miRNA) which may post-transcriptionally regulate as many as 60% of human genes [104], have also been performed during prion disease pathogenesis, and have identified potentially dysregulated signaling pathways [100, 105, 106]. However, the biological roles of most miRNAs have yet to be characterized, which makes it difficult to interpret the effects mediated by many of the identified miRNAs.

The kinomics approach described here of course has limitations, too. First, the primary screens identify signaling pathways of potential interest using hierarchical clustering. The identification of potentially dysregulated pathways requires multiple kinases in the pathway to be included in the primary screen. The 137 protein kinases selected for the screens are involved in most of the best characterized signaling pathways. The approach is thus unable to detect signaling pathways that are less well characterized, or for which only one or very few kinases are included in the primary screen. Protein kinases are also extensively regulated post-translationally. Dysregulation of signaling pathways during chronic conditions often result in, or is the result of, changes in expression levels of involved proteins. We therefore screened for expression levels in the primary analysis. Consequently, these screens cannot detect pathways that are only affected post-translationally (unless the posttranslational regulation is at the level of degradation). The described approach is also unable to differentiate signaling pathways dysregulated as a result of disease from those critical in the pathogenesis. Previous studies have screened protein kinase inhibitors and identified STI571 (an inhibitor of c-Abl, c-Kit, and PDGFR kinases) as promoting PrP^Sc^ degradation [20]. The signaling pathway identified by the kinomics approach can be tested with siRNA, knockouts, or specific inhibitors, to identify whether it is involved in CyPrP pathogenesis, or dysregulated as a result of it.

## Conclusions

We used N2a cells expressing cytoplasmic PrP mutants to test the sensitivity and specificity of kinomics analyses developed to detect the dysregulation of specific signaling pathways. The assays identified a dysregulated signaling pathway which is fully consistent with previous data. We conclude that the kinomics analyses are sensitive and specific enough to detect signaling pathways dysregulated in a simple *in vitro* model of prion pathogenesis. We have now used these analyses to test critical signaling pathways dysregulated in brains of prion-infected animals (see companion paper).

## Materials and methods

### Cloning, cell culture and transfections

Cloning of CyPrP^EGFP^, CyPrP^EGFP^124stop and CyPrP^EGFP^124-230 in pCEP4β (Life Technologies Inc., Carlsbad, California, USA) was previously described [56]. Mouse N2a neuroblastoma cells were maintained in Dulbecco’s Modified Eagle’s Medium (DMEM) supplemented with 10% fetal bovine serum (Wisent, St. Bruno, Quebec, Canada). Transfections were carried out using Exgen (MBI Fermentas, Burlington, Ontario, Canada) or GeneCellin (BioCellChallenge, Toulon, France), according to the manufacturer’s protocol. To evaluate transfection efficiency, cells were harvested with trypsin and EDTA and centrifuged for 5 min at 500 × *g*. Following a wash with PBS, cells were fixed with 4% paraformaldehyde in 4% sucrose for 20 min at room temperature, washed with PBS and analyzed on a cytometer for GFP expression.

Mouse 3T3 cells were propagated in DMEM (Life Technologies Inc.) supplemented with 5% fetal bovine serum (FBS; PAA Laboratories GmbH, Pasching, Austria), 50 U/mL penicillin and 50 U/mL streptomycin (Life Technologies Inc.), at 37°C in 5% CO_2_.

### Cell lysis and brain homogenization

Mouse 3T3 and transfected N2a cells were cultured to approximately 85% confluency on 8 × 10 cm tissue culture dishes. All subsequent procedures were performed on ice or at 4°C using reagents pre-chilled to 4°C. Each dish was washed twice with 2 mL of phosphate-buffered saline (PBS; 150 mM NaCl, 1 mM KH_2_PO_4_, 3 mM Na_2_HPO_4_, pH 7.4). Cells were collected by scraping into freshly prepared lysis buffer (0.2 mL per dish) (20 mM MOPS [pH 7.0)], 2 mM EGTA, 5 mM EDTA, 1% Nonidet P-40, 0.001% phosphatase inhibitor cocktail [Pierce, Rockford, Illinois, USA], 0.002% protease inhibitor cocktail [Sigma-Aldrich, St. Louis, Missouri, USA], 1 mM DTT, pH 7.2). The lysates were passed twice through a 20 gauge needle, sonicated five times for 20 s intervals at 88 W output (XL-2020; Misonix, Farmingdale, New York, USA), and pre-cleared at 14,000 × *g* for 30 min (JA.14 rotor, Avanti J-E centrifuge; Beckman/Coulter, Brea, California, USA). Approximately 1 mL volumes of supernatant were aliquoted, snap frozen in liquid nitrogen, and immediately stored at −80°C (3T3 lysates) or shipped on dry ice and then stored at −80°C (N2a lysates).

Brain homogenates were prepared using snap-frozen mouse brains stored at −80°C. Weighed brains were homogenized in 3 mL freshly prepared lysis buffer per 250 mg of brain, using a tissue homogenizer (TH; OMNI International, Kennesaw, Georgia, USA) with disposable tips (hard tissue OMNI tip; OMNI International). Homogenates were then sonicated, centrifuged, and stored as described for cell lysates.

### Protein quantitation

Protein concentration was determined by Bradford’s assay (Bio-Rad Laboratories, Hercules, California, USA). Protein concentration and equal sample loading were then verified by preliminary sodium dodecyl sulfate polyacrylamide gel electrophoresis (SDS-PAGE). Brain homogenates or cell lysates were mixed with equal volumes of 2X SDS loading buffer (125 mM Tris-Cl [pH 6.8], 20% glycerol, 4% SDS, 0.005% bromophenol blue, 260 mM DTT), denatured at 100°C for 10 min, and loaded onto 10- or 15-well 8% SDS-PAGE gels (Mini-PROTEAN; Bio-Rad Laboratories) (running buffer; 190 mM glycine, 24.8 mM Tris, 0.1% SDS, pH 8.3). Proteins were run through the stacking gel at 50 V, and then resolved for 90 min at 100 V, always at room temperature. Proteins were stained with Coomassie blue G-250 (Bio-Safe Coomassie; Bio-Rad Laboratories) according to the manufacturer’s instructions. Signal from Coomassie-stained protein was detected using an Odyssey infrared imaging system (LI-COR Biosciences, Lincoln, Nebraska, USA) in the 700 nm channel and quantitated using Odyssey 3.0 software (LI-COR Biosciences). Protein amounts were calculated relative to a pre-quantitated standard brain homogenate.

### Western blot

All procedures were performed at room temperature and all washes used gentle rocking. Proteins were denatured by incubating for 10 min at 100°C with an equal volume of 2X, or one-fifth volume of 6X (375 mM Tris-Cl [pH 6.8], 60% glycerol, 12% SDS, 0.015% bromophenol blue, 780 mM DTT), SDS-PAGE loading buffer.

For Western blots of PrP and EGFP, lysates from N2a cells transfected with empty vector (100 μg), or vector encoding EGFP (50 μg), CyPrP^EGFP^ (200 μg), CyPrP^EGFP^124stop (100 μg), or CyPrP^EGFP^124-230 (100 μg) were loaded onto 15-well 10% SDS-PAGE gels (Mini-PROTEAN; Bio-Rad Laboratories). Proteins were resolved as described for protein quantitation and the gels were then equilibrated in transfer buffer (384 mM glycine, 49.6 mM Tris, 20% methanol, 0.01% SDS) [107] for 30 min. Meanwhile, polyvinylidene fluoride (PVDF) membranes (Immuno-Blot, 0.2 μm; Bio-Rad Laboratories) were soaked in methanol for 2 min, and equilibrated in transfer buffer for 20 min. For each membrane, four sheets of filter paper were equilibrated in transfer buffer for 5 min. Transfer cassettes were loaded into a transfer tank (TE22; Hoefer, Holliston, Massachusetts, USA) filled with transfer buffer at 4°C. Proteins were transferred for 23 h at 4°C; 1 h at 54 mA, 4 h at 189 mA, 8 h at 270 mA and finally 10 h at 378 mA. The temperature was maintained at 4°C by heat exchange (Isotemp 1016D; Thermo Fisher Scientific, Waltham, Massachusetts, USA) and gentle stirring of the transfer buffer. After transfer, membranes were dried, soaked in methanol for 2 min and then washed twice for 10 min each in Tris-buffered saline (TBS; 140 mM NaCl, 3 mM KCl, 25 mM Tris, pH 7.6). Membranes were blocked for 1 h in 10% blocking buffer (Sigma-Aldrich) then simultaneously probed for 18 h at 4°C with primary antibodies specific for GFP (a kind gift from Dr. Luc Berthiaume, University of Alberta) and PrP (clone 3F4; a kind gift from Dr. Deborah McKenzie, University of Alberta) diluted to 1:10,000 and 1:2,500, respectively, in 10% blocking buffer with 0.1% Tween-20. Afterward, membranes were washed in TBS with 0.1% Tween-20 (TBST) once for 5 min and thrice for 10 min each. Membranes were incubated with donkey anti-mouse IRDye 680- and donkey anti-rabbit IRDye 800-labeled secondary antibodies (LI-COR Biosciences), diluted to 1:20,000 in 10% blocking buffer with 0.1% Tween-20 and 0.01% SDS for 1 h. Membranes were washed thrice in TBST for 10 min each and once in TBS for 5 min. Signal from pre-stained protein standards and IRDye 680-labeled secondary antibody was detected at 700 nm, and from IRDye 800-labeled secondary antibody at 800 nm, using an Odyssey infrared imaging system. Signal was quantitated using Odyssey 3.0 software. To evaluate the amounts of protein transferred to the membranes, the membranes were stained with Coomassie blue R-250 (Bio-Rad Laboratories) for 10 min and destained with 40% methanol in 10% glacial acetic acid thrice for 10 min each, or until excess stain was removed. Signal from Coomassie-stained protein was detected at 700 nm using the Odyssey and quantitated using Odyssey 3.0 software.

For multiplex Western blot spiking experiments, 85 μg (for p39 and TrkB) or 200 μg (for PKCγ, CaMK4, CDK1, CHK1, RSK2, CDK4, PDGFRβ, BTK and PKCθ) of total protein was loaded per linear cm of four- or five-well 8% SDS-PAGE gels. For primary multiplex Western blots of N2a cells expressing cytoplasmic PrP mutants, 150 μg of protein was loaded per linear cm of single-well 8% SDS-PAGE gels; 8 or 10% gels, as appropriate for the target MW, loaded with 150, 300, or 450 μg of protein per linear cm were used for targeted analyses. Similar signal intensities were reached using 150 μg of N2a or 200 μg of brain lysates (Additional file 4: Figure S3). Proteins were resolved and transferred to PVDF membranes as described for Western blots of EGFP or CyPrP^EGFP^. Membranes were blocked for 1 h in 10% blocking buffer (Sigma-Aldrich) for protein-specific antibodies, or in 3% BSA (Rockland, Gilbertsville, Pennsylvania, USA) for phosphorylation-specific antibodies. Membranes were rinsed briefly with TBS and positioned within a 20-lane multi-screen apparatus (Bio-Rad Laboratories, Hercules, California, USA). Meanwhile, combinations of primary antibodies were diluted in 2.6 mL (primary blots) or 3.9 mL (spiked, secondary and tertiary blots) of 10% blocking buffer or 3% BSA with 0.1% Tween-20. Six hundred microliters of antibody solutions were loaded in each lane of the multi-screen apparatus and incubated for 18 h at 4°C. Membranes were then briefly washed once with TBST within the multi-screen apparatus, removed from the apparatus and further washed in TBST, once for 5 min and four times for 15 min each. Membranes were incubated with secondary antibody diluted to 1:20,000 in 10% blocking buffer or 3% BSA with 0.1% Tween-20 and 0.01% SDS for 1 h. Mouse monoclonal primary antibodies were detected with goat anti-mouse Alexa Fluor (Molecular Probes, Eugene, Oregon, USA) or IRDye (LI-COR Biosciences) 680-labeled secondary antibody. Rabbit or goat polyclonal primary antibodies were detected with goat anti-rabbit or donkey anti-goat (LI-COR Biosciences or Rockland) IRDye 800-labeled secondary antibodies, respectively. Signal from pre-stained protein standards and Alexa Fluor or IRDye 680-labeled secondary antibody was detected at 700 nm, and from IRDye 800-labeled secondary antibody at 800 nm, using the Odyssey system. Signal was quantitated using Odyssey 3.0 software.

Membranes with protein from cells expressing cytoplasmic PrP mutants were always stripped (only once) in parallel with the membranes from the control cells, under conditions that minimize protein loss [108]. Membranes were washed with a mild stripping buffer (25 mM glycine, 1% SDS, pH 2.0) once for 5 min and twice for 15 min each. Membranes were then washed with TBST once and TBS once for 5 min each. If necessary, they were further washed with mild stripping buffer for a maximum of six times of 15 min each. If still necessary, membranes were then incubated with harsh stripping buffer (50 mM Tris-Cl [pH 7.0], 2% SDS, 50 mM DTT) [109] for 15 min at 65°C with gentle rocking. Membranes were then washed with TBST once for 5 min and TBS twice for 10 min each. Stripped membranes were blocked and reprobed with another set of primary antibodies as described. No membrane was stripped more than once.

### Hierarchical cluster analysis

The integrated intensity levels (pixel volume) of proteins in cells expressing cytoplasmic PrP mutants were normalized to levels in cells expressing empty vector, and then log_2_ transformed to avoid bias by proteins with extreme changes in expression. Hierarchical clustering was performed with Gene Cluster 3.0 [110] using city-block distance (similarity metric) and complete linkage (clustering method). Java Treeview was used to present the resulting clusters [111].

### Time-course data normalization

To evaluate the changes in protein and phosphorylation levels in cells actually expressing CyPrP^EGFP^, the raw levels (in the population containing expressing and non-expressing cells) were corrected for differences in transfection efficiency in each biological replicate and then expressed as relative to the levels in cells transfected with the EGFP expression construct using Equation 1.1

Where,

G_i_: pixel volume in lysates from cells expressing EGFP in replicate “*i*”,

P_i_: pixel volume in lysates from cells expressing CyPrP^EGFP^ in replicate “*i*”, and

TE: transfection efficiency.

The differences in the integrated intensity levels (pixel volume) in the populations of cells transfected with the vectors expressing CyPrP^EGFP^ or EGFP from the same biological replicate were corrected by the fraction of cells actually expressing the recombinant proteins (transfection efficiency, 47, 45 and 34% for replicates 1, 2 and 3, respectively), which are the cells in which the expressed proteins directly induce changes in signaling, and added to the basal levels (those in the population of cells transfected with the vector expressing EGFP). The corrected levels in cells expressing CyPrP^EGFP^ were then normalized to the levels in cells expressing EGFP in the same biological replicate.

### Statistical analyses

All data was analyzed using Prism (Version 5.0f; GraphPad Software Inc., La Jolla, California, USA). For nonlinear regression analyses, curves of the normalized phosphorylation levels were compared to a straight line (y-intercept = 1; slope = 0), representing the levels in cells expressing EGFP, using a replicates test for lack-of-fit.

## Electronic supplementary material

Additional file 1: Figure S1: The protein kinases selected for primary multiplex Western blots represent all major groups of the human protein kinases. The human kinome, the protein kinase complement of the human genome, clustered by protein kinase domain homology (modified from Manning *et al.,* 2002 [50]) The 145 protein kinases initially selected for analyses are outlined in red. ATM, which is a member of the atypical group of protein kinases, does not cluster with any group, and is therefore not presented. (PPT 4 MB)

Additional file 2: Table S1: Accession number and antibody source for each of the 127 protein kinases and 10 regulatory subunits optimized for analyses in primary multiplex Western blots. One hundred and twenty-two protein kinases or regulatory subunits included in the multiplex Western blots were detected in 200 μg of mouse brain homogenate per linear well cm. The other 15 (indicated by the asterisks) were detected in multiplex Western blots using an equivalent amount of cell lysate from cycling 3T3 mouse fibroblasts. The human accession number and source of the antibody used for each protein are indicated. (XLS 45 KB)

Additional file 3: Figure S2: Western blot for cytoplasmic PrP mutants in N2a cells. Protein from N2a cell lysates transfected with empty vector, or vector encoding CyPrP^EGFP^ (CyPrP), CyPrP^EGFP^124stop (124stop), or CyPrP^EGFP^124-230 (124-230) was resolved, transferred to membranes and probed with α-PrP (which recognizes an epitope in residues 109-112) and α-GFP antibodies. Molecular weights in kDa are indicated to the right. The arrowheads to the left indicate the molecular weight of CyPrP^EGFP^ (48 kDa), CyPrP^EGFP^124stop (38 kDa), and CyPrP^EGFP^ 124-230 (34 kDa). Asterisks indicate specific bands. CyPrP^EGFP^ and CyPrP^EGFP^124stop were detected by α-PrP and α-GFP antibodies, CyPrP^EGFP^ 124-230, which does not have the epitope recognized by the α-PrP antibody, was recognized only by the α-GFP antibody. A background band with a molecular weight close to that of CyPrP^EGFP^ cross-reacted with the α-GFP antibody. Membranes were stained with Coomassie to analyze total protein. Dashed lines separate different blots. (PPT 1 MB)

Additional file 4: Figure S3: Frequency distribution of signal intensity in N2a and mouse brain lysates. Signal for each protein kinase detected was quantitated after multiplex Western blots with 200 μg of mouse brain or 150 μg of N2a cell lysate per linear centimeter of gel. The number of protein kinases yielding signal intensities in each range is plotted. The frequency distribution of the signal intensity in both lysates is highly similar. (PPT 152 KB)
