# Supplementary figures and images for: Development of kinomic analyses to identify dysregulated signaling pathways in cells expressing cytoplasmic PrP
Source: Virol J. 2014 Oct 3;11:175. doi: 10.1186/1743-422X-11-175 (PMC4283144; doi:10.1186/1743-422X-11-175)

## Slide 1
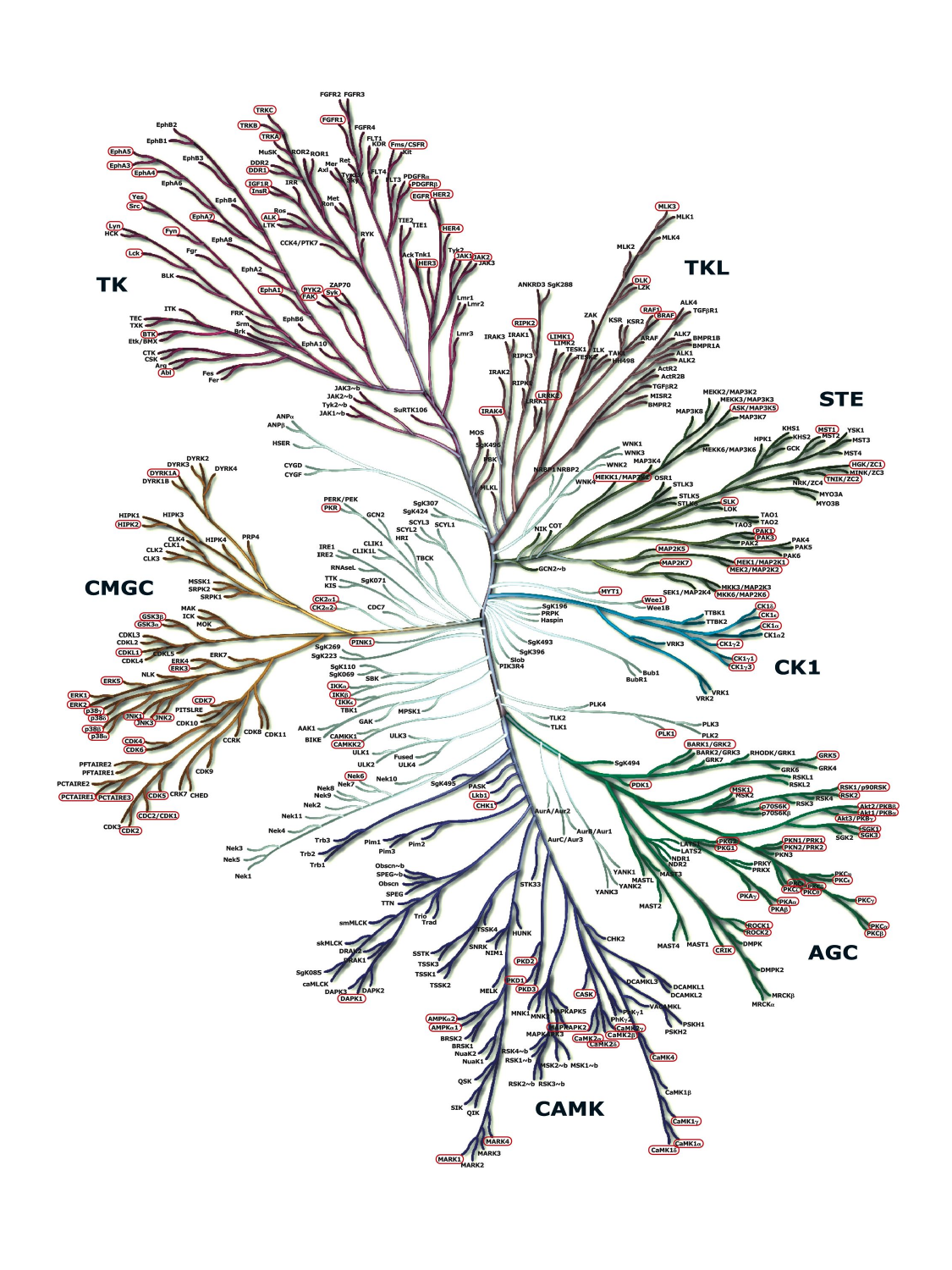

Supplement: Supplementary file 1 — Additional file 1: Figure S1: The protein kinases selected for primary multiplex Western blots represent all major groups of the human protein kinases. The human kinome, the protein kinase complement of the human genome, clustered by protein kinase domain homology (modified from Manning et al., 2002 [50]) The 145 protein kinases initially selected for analyses are outlined in red. ATM, which is a member of the atypical group of protein kinases, does not cluster with any group, and is therefore not presented. (PPT 4 MB) [file 12985_2014_2533_MOESM1_ESM.ppt]

## Slide 1
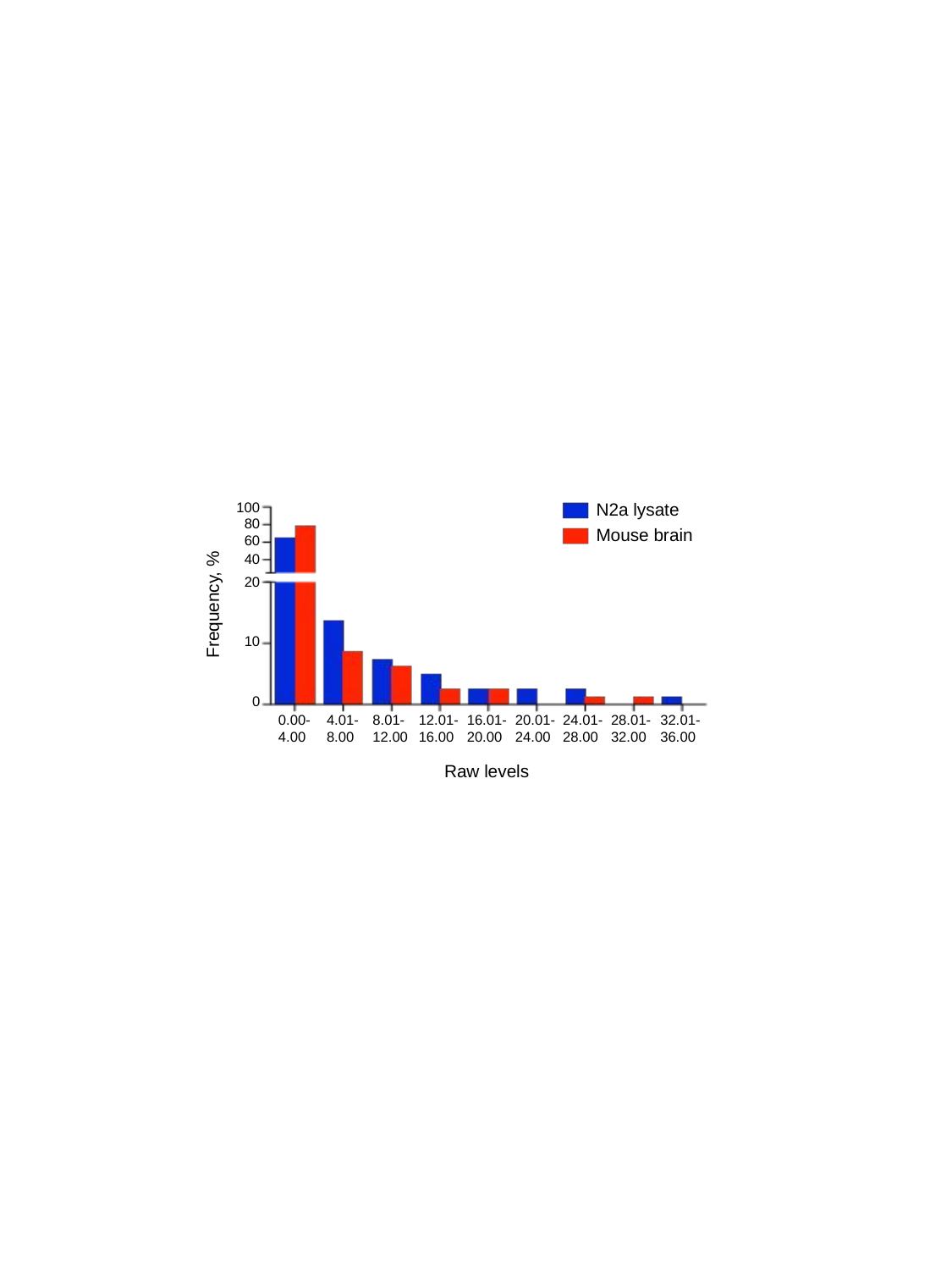

N2a lysate
Mouse brain
100
80
60
40
20
Frequency, %
10
0
0.00-
4.00
4.01-
8.00
8.01-
12.00
12.01-
16.00
16.01-
20.00
20.01-
24.00
24.01-
28.00
28.01-
32.00
32.01-
36.00
Raw levels

Supplement: Supplementary file 4 — Additional file 4: Figure S3: Frequency distribution of signal intensity in N2a and mouse brain lysates. Signal for each protein kinase detected was quantitated after multiplex Western blots with 200 μg of mouse brain or 150 μg of N2a cell lysate per linear centimeter of gel. The number of protein kinases yielding signal intensities in each range is plotted. The frequency distribution of the signal intensity in both lysates is highly similar. (PPT 152 KB) [file 12985_2014_2533_MOESM4_ESM.ppt]
