# Supplementary material for: Development of kinomic analyses to identify dysregulated signaling pathways in cells expressing cytoplasmic PrP
Source: Virol J. 2014 Oct 3;11:175. doi: 10.1186/1743-422X-11-175 (PMC4283144; doi:10.1186/1743-422X-11-175)

## Slide 1
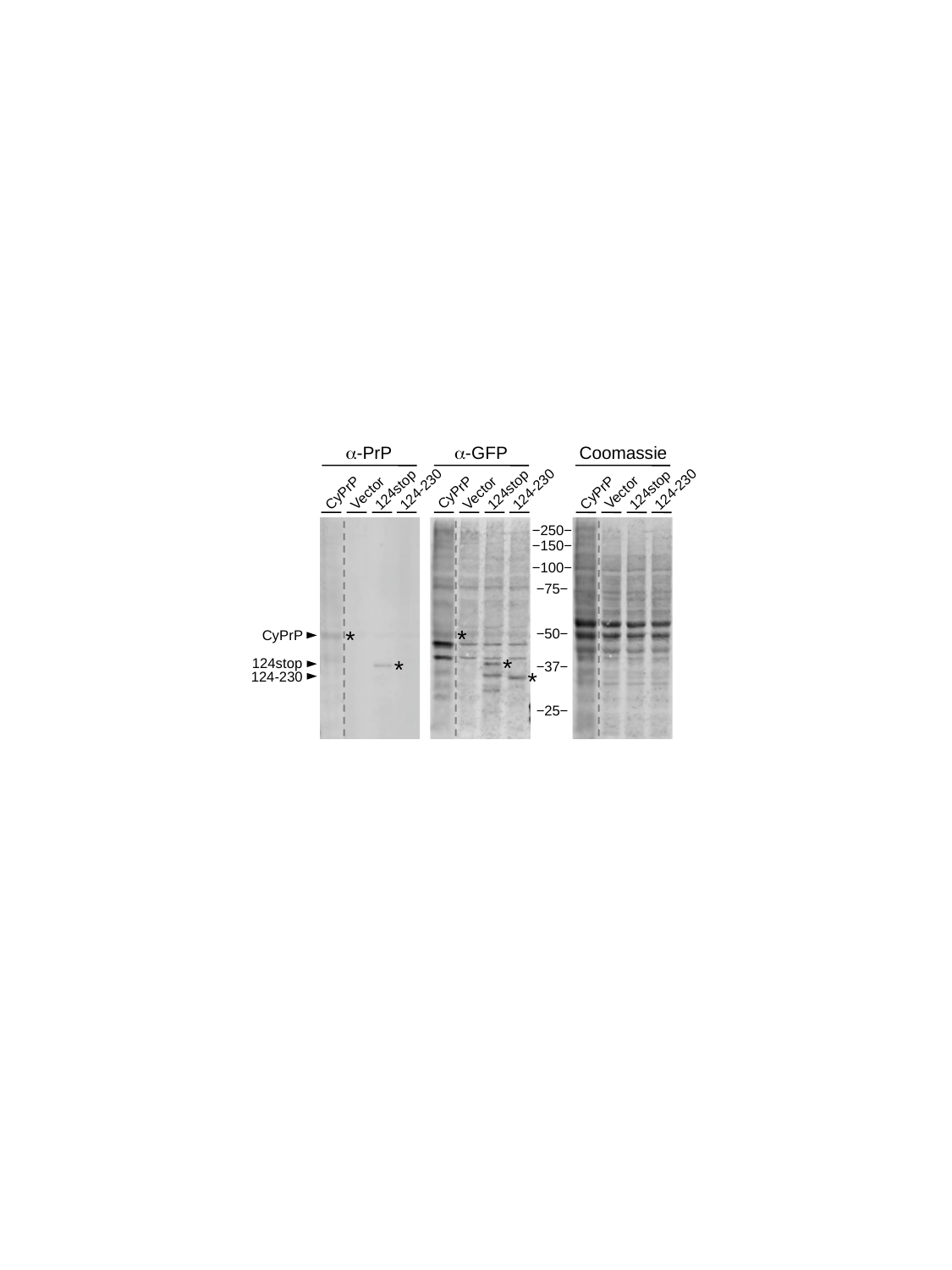

α-PrP
124-230
124stop
CyPrP
Vector
α-GFP
124-230
124stop
CyPrP
Vector
Coomassie
124-230
124stop
Vector
CyPrP
−250−
−150−
−100−
−75−
−50−
−37−
−25−
*
*
CyPrP
*
*
124stop
*
124-230

## Slide 2
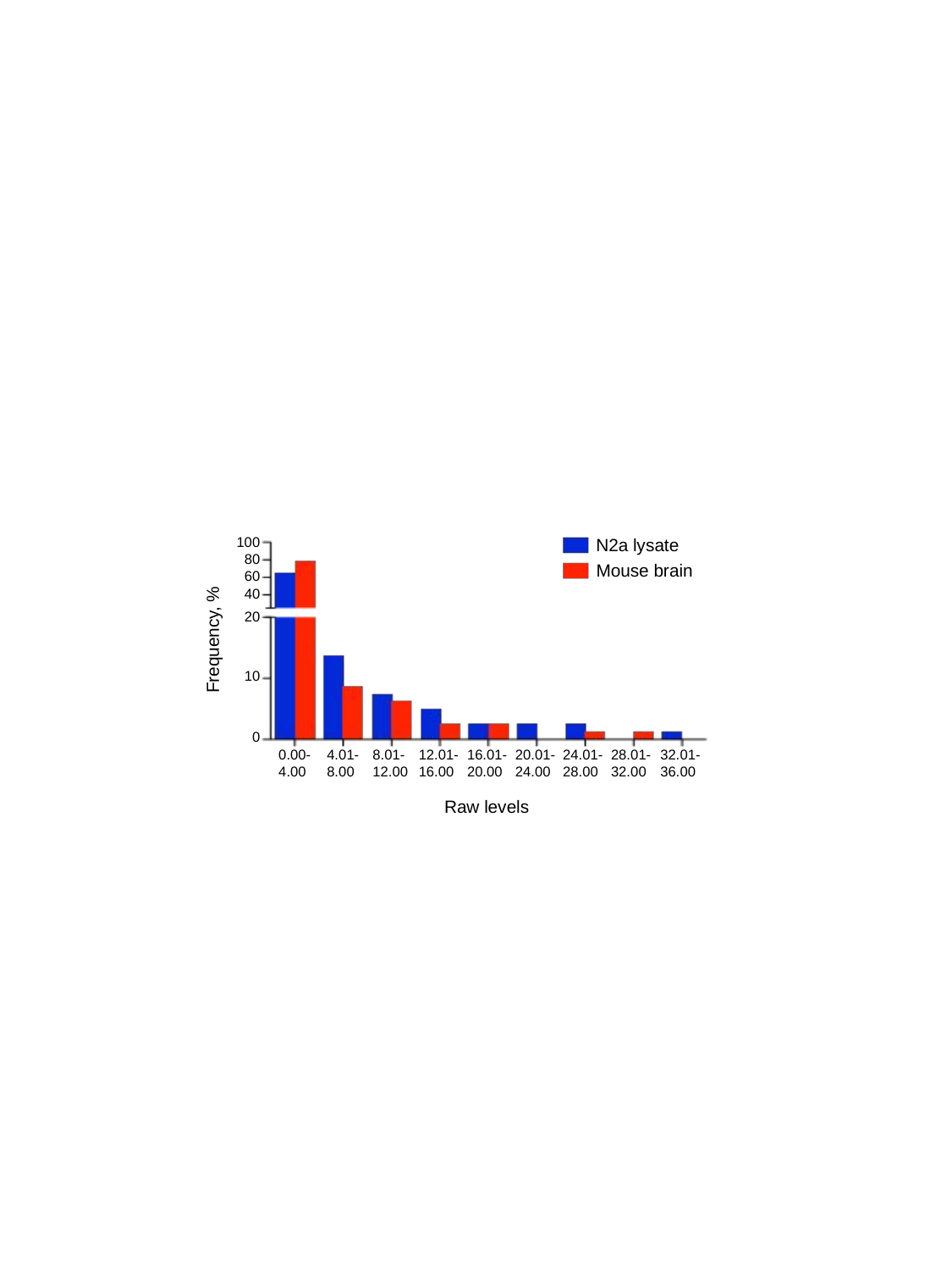

N2a lysate
Mouse brain
100
80
60
40
20
Frequency, %
10
0
0.00-
4.00
4.01-
8.00
8.01-
12.00
12.01-
16.00
16.01-
20.00
20.01-
24.00
24.01-
28.00
28.01-
32.00
32.01-
36.00
Raw levels

## Slide 3
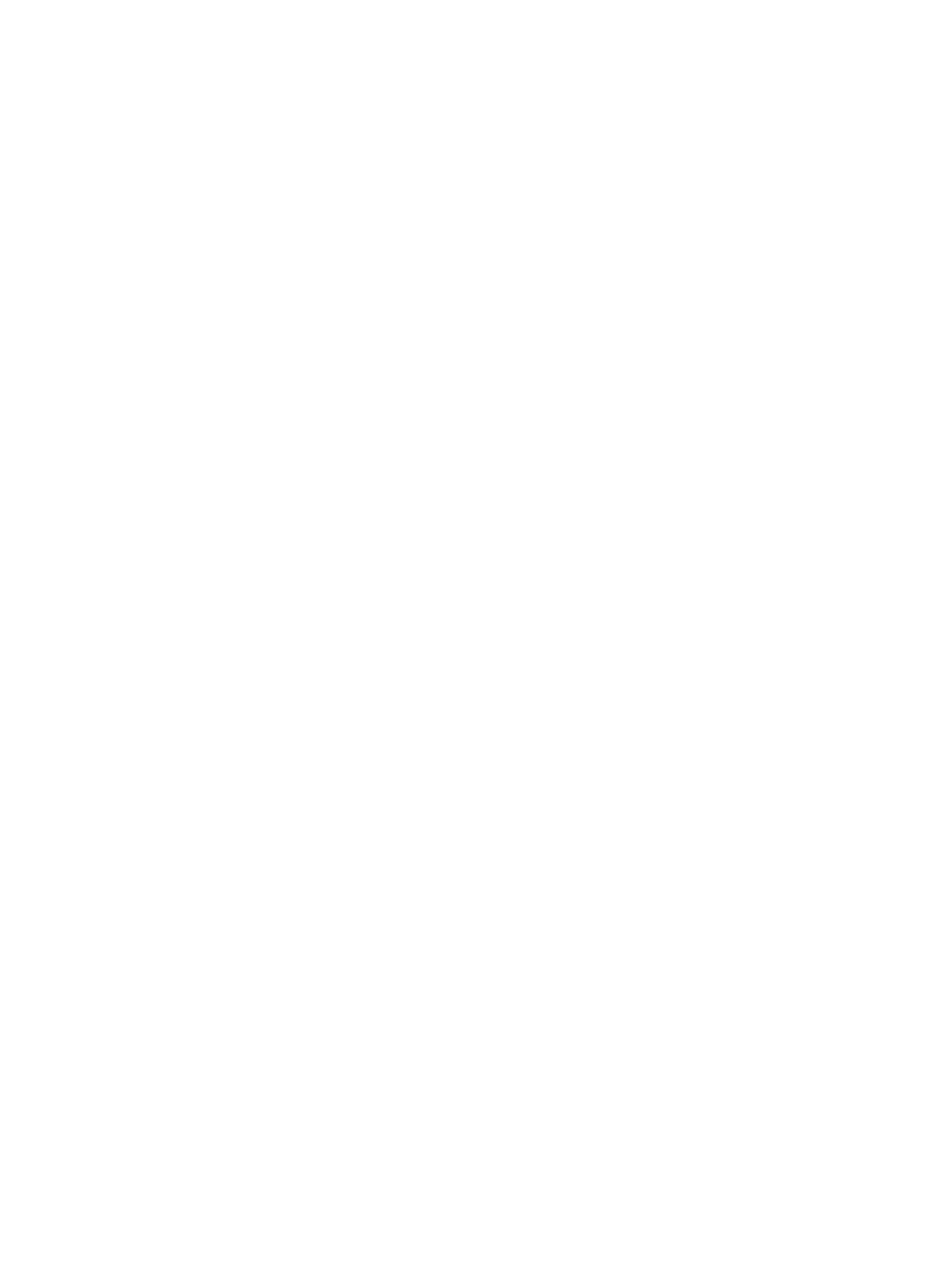

Supplement: Supplementary file 3 — Additional file 3: Figure S2: Western blot for cytoplasmic PrP mutants in N2a cells. Protein from N2a cell lysates transfected with empty vector, or vector encoding CyPrPEGFP (CyPrP), CyPrPEGFP124stop (124stop), or CyPrPEGFP124-230 (124-230) was resolved, transferred to membranes and probed with α-PrP (which recognizes an epitope in residues 109-112) and α-GFP antibodies. Molecular weights in kDa are indicated to the right. The arrowheads to the left indicate the molecular weight of CyPrPEGFP (48 kDa), CyPrPEGFP124stop (38 kDa), and CyPrPEGFP 124-230 (34 kDa). Asterisks indicate specific bands. CyPrPEGFP and CyPrPEGFP124stop were detected by α-PrP and α-GFP antibodies, CyPrPEGFP 124-230, which does not have the epitope recognized by the α-PrP antibody, was recognized only by the α-GFP antibody. A background band with a molecular weight close to that of CyPrPEGFP cross-reacted with the α-GFP antibody. Membranes were stained with Coomassie to analyze total protein. Dashed lines separate different blots. (PPT 1 MB) [file 12985_2014_2533_MOESM3_ESM.ppt]
